# A Case Report of Appendicitis Causing Cecal Volvulus: A Rare Occurrence

**DOI:** 10.7759/cureus.58505

**Published:** 2024-04-18

**Authors:** Bhovineey Ramanathan, Vinod Ramachandran, Kimberley Tan

**Affiliations:** 1 Department of Surgery, Lyell McEwin Hospital, Adelaide, AUS

**Keywords:** appendix, operation, abdomen, volvulus, cecal, appendicitis

## Abstract

This case report presents a fascinating scenario involving a 60-year-old female who was diagnosed with cecal volvulus secondary to appendicitis. The patient’s initial presentation included a three-day history of periumbilical pain accompanied by reduced oral intake and an inability to pass stool. Through a systematic approach involving detailed history-taking, comprehensive physical examinations, and pertinent imaging studies, a precise diagnosis of cecal volvulus induced by appendicitis was established. Subsequently, the patient underwent a timely operation, leading to a successful resolution of her condition and a remarkably swift recovery post-surgery.

This unique case prompts a deeper exploration into the incidence and management of this rare phenomenon, where the seemingly unrelated condition of appendicitis precipitated a cecal volvulus. Given the unusual nature of this presentation, it underscores the importance of considering atypical etiologies in patients presenting with signs and symptoms of bowel obstruction. This discussion aims to shed light on the diagnostic challenges, treatment strategies, and outcomes associated with this intriguing interplay of pathologies, offering valuable insights for clinicians encountering similar cases in their practice.

## Introduction

Cecal volvulus, a rare but serious medical condition, entails the twisting or rotation of the mobile cecum and often involves the ascending colon as well [1]. This condition is relatively uncommon, with an estimated occurrence of approximately 2.8 to 7.1 cases per million people annually, representing roughly 1% of all cases of intestinal obstruction [2]. Interestingly, cecal volvulus appears to be more prevalent in Western countries. Several established risk factors contribute to the likelihood of experiencing this condition, including advanced age, chronic constipation, diets high in fiber, pregnancy, and a history of prior abdominal surgeries [3]. These factors collectively underscore the importance of vigilance and preventive measures in susceptible populations.

## Case presentation

A 63-year-old female patient who lived at home with her husband presented with a three-day history of abdominal pain and vomiting. The pain was mainly focussed on the epigastric and umbilicus region. She described it to be a constant sharp pain that worsened over time. Reportedly, she also had a reduced oral intake and appetite with not being able to open her bowels for the same period of time. Otherwise, she denied having fever, urinary changes, sick contacts, respiratory symptoms, or any other constitutional symptoms. She reported that she had never had similar pain in the past and was unsure what would have caused it.

Regarding her past medical history, she had autism, for which she was on a disability pension, depression, anxiety, irritable bowel syndrome, gastroesophageal reflux disease, and chronic neck pain. Her only surgical history was two previous cesarean sections. Her only regular medication was an antidepressant, amitriptyline. She was an active smoker of 15 pack years and only claimed to drink alcohol occasionally. Her body mass index was around 25 kg/m^2^.

The patient was initially referred by the emergency department doctor to the surgical team with an acute abdomen with only baseline blood but no imaging available. Due to the acuity, the patient was immediately attended to in the emergency department by the surgical team.

Physical examination

She had a Glasgow Coma Scale score of 15. Her vitals on arrival were within normal limits. On examination, the surgical team noted that she was tender on both her epigastric and umbilicus with mild tenderness over the right iliac fossa as well. There was no rebound tenderness and nil peritonism was noted. There was no obvious respiratory distress but the patient was experiencing shallow breathing due to her abdominal pain. The rest of the physical examination was unremarkable.

Laboratory investigations

Her initial baseline blood revealed leucocytosis with elevated inflammatory markers and an acute kidney injury likely due to dehydration from the constant vomiting. At this point, a decision was made to proceed with a CT of the abdomen to rule out differentials such as acute cholecystitis, mesenteric adenitis, appendicitis, pancreatitis, and other possible intra-abdominal causes. She was immediately started on normal saline hydration and kept fasting. Mild analgesics in terms of oxycodone were given to control her pain and ondansetron to prevent vomiting.

Imaging

**Figure 1:**
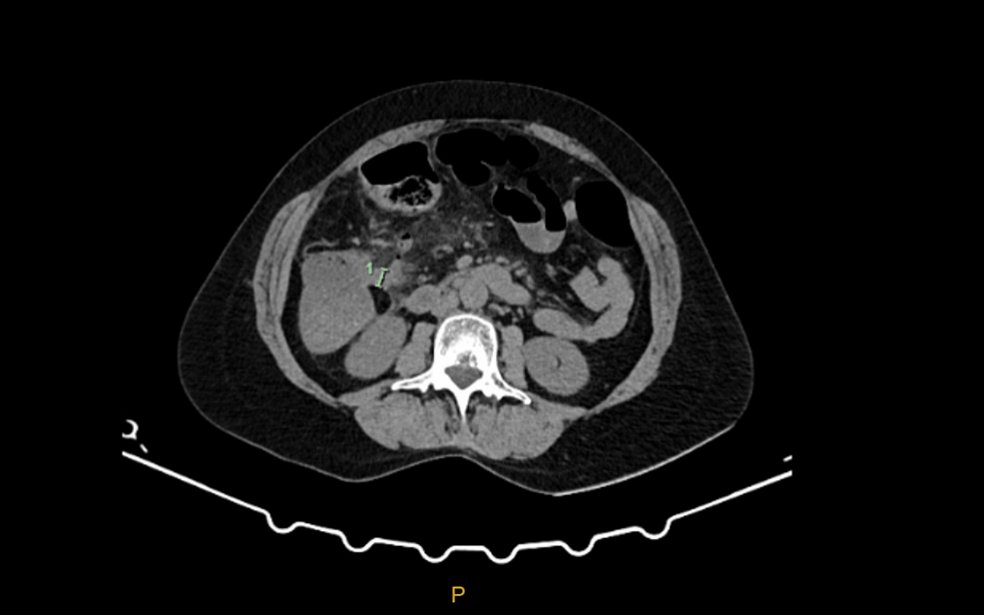
CT scan axial view showing the base of the cecum and appendix with surrounding inflammation.

**Figure 2:**
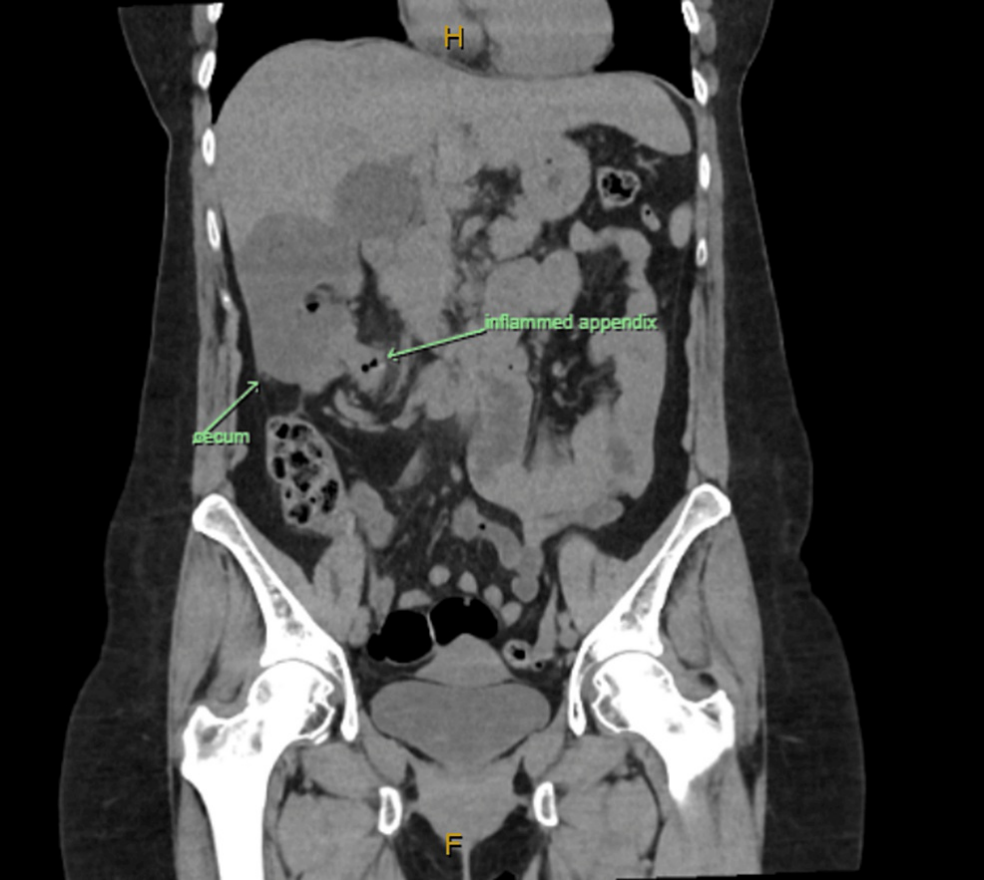
Coronal view showing the inflamed appendix and position of the cecum in the right upper quadrant.

The CT of the abdomen (Figures 1, 2) revealed a rotated cecum with the cecal pole lying inferior to the liver and expected hepatic flexure. There was evidence of appendicitis (periappendiceal stranding and thickened appendiceal wall), with the appendix being located in the mid to upper right abdomen anterior to the lower pole of the right kidney and lateral to the second/third part of the duodenum region. There was reactive lymphadenopathy and stranding in the mesentery but no perforation or abscess collection was seen.

The patient was started on triple antibiotics (gentamicin, amoxicillin, and metronidazole) and a decision was made to proceed with appendicectomy and possible right hemicolectomy based on the clinical findings and imaging.

Operative approach and findings

Intraoperatively, it was noted that the appendix was perforated at the base and there was localized purulent peritonitis in the right upper quadrant. The appendiceal inflammation point was noted to be the point of rotation for cecal volvulus. There was no evidence of bowel ischemia secondary to the volvulus. The colon was de-volved into its anatomical position after dissecting the adhesions.

A right hemicolectomy was performed and a complete run of the bowel from the duodenojejunal flexure to the splenic flexure was done to confirm the appropriate anatomical position. A 19-Fr drain was placed. Postoperative orders include sips of water overnight with a view of starting clear fluids on review mane, drain monitoring, and continuing intravenous antibiotics that had been started.

Postoperative recovery and discharge

The patient had a speedy recovery with the drain being able to be removed on day two, the patient achieving proper ambulation by day three, bowels opened by day four, and pain controlled by day five postoperatively. The patient was discharged on day five postoperatively after ensuring the blood results were trending down in terms of the inflammatory markers and the patient was confident enough to cope at home with minimal discomfort. An outpatient appointment for review in three weeks was given before discharge.

Histopathology review

The sample obtained from the right hemicolectomy was sent for histopathology. It revealed a gangrenous appendix with perforation and active serositis. There was adherent fibrinous exudate around the serosa of the cecum and appendix. A perforation measuring 4 mm in diameter was seen at the base of the appendix 20 mm from the appendiceal opening. The bowel mucosa otherwise appeared normal. There was no dysplasia or malignancy in the sections examined.

## Discussion

Appendicitis causing cecal volvulus and its description in the literature is very rare. It was first described by Cochrane in 1929 [4]; however, there have been fewer than 10 articles in total that explain the phenomena. The latest article was published in Pakistan (Lahore) [5] of a 55-year-old female presenting with a three-day history of umbilical pain.

Volvulus occurs when there is a twisting of mesentery around an axis. Cecal volvulus can only occur when the posterior parietal peritoneum is incompletely fused with the right colon (an embryological variant present in 10-37% of adults). In 1938, Weinstein described a condition known as a cecal bascule. Cecal bascule [6] describes when the volvulus involves the cecum alone and occurs due to the transposition of the mobile cecum from a caudal to a cephalad direction. It has been noted that cecal volvulus [7] predominantly occurs between the ages of 20-30 years. There are three different types of cecal volvulus. Type 1 involves a clockwise axial torsion or twisting of the cecum along the long axis when the location is usually in the right lower quadrant. Type 2 develops from torsion or twisting of the cecum [8] and a part of the terminal ileum, the location of the cecum gets displaced to an ectopic position (as seen in our patient when it is present in the left upper quadrant) and is relocated in an inverted orientation. Type 3, which is known as the cecal bascule, is the upward folding of the cecum with no axial twisting. The majority of the volvulus [9] belong to Type 1 and Type 2 (80%).

The clinical presentation [10] is variable as it ranges from intermittent abdominal pain to persisting severe abdominal pain due to intestinal ischemia and necrosis. Pain is usually associated with vomiting, occasionally bilious. The recurrent intermittent pattern of cecal volvulus, known as mobile cecum syndrome, occurs in nearly 50% of patients before the onset of acute volvulus.

Patients suspected of a volvulus should have a suitable radiographic workup. Plain film abdominal X-rays are only diagnostic in a limited number of cases where the classic “coffee bean” sign point to the left upper quadrant is apparent. This is usually accompanied by paucity of rectal gas. CT is the gold standard [9] in assessing the bowel for signs of possible ischemia where pneumatosis, abdominal free fluid, intestinal thickening, and bowel wall hypoenhancement are indications for urgent operative management.

Some others have reported successful reduction [11] of cecal volvulus with colonoscopy. However, the success rate is only 30% and endoscopy decompression is not advised to be attempted in cecal volvulus due to inefficacy and its potential harm; hence, urgent surgical treatment with ileocolic resection [7] and anastomosis or cecopexy is preferred in stable patients with a viable cecum. Ileocecal resection is required in cases of complicated cecal volvulus with abscess, perforation, and hemodynamic instability. Performing only a reduction of the volvulus [12] is associated with a high recurrence rate. Apart from that, there is conflicting evidence on the advocation of cecopexy vs. ileocolic resection/hemicolectomy. Some studies strongly agree that resection should be performed in all cases of cecal volvulus regardless of the viability of the cecum, while others claim that resection would increase the rate of morbidity and mortality compared to cecopexy. A study [13] that followed 50 patients in the long term by O’Mara et al. noted that 18 of the patients who underwent cecopexy had no operative deaths and minimal postoperative complications. There were no recurrences in these 18 patients during the five-year follow-up. The study concluded the safety of cecopexy. However, another study [14] noted that ileocolic resection is the standard treatment for cecal volvulus with a low rate of mortality and 0% rate of recurrence.

Cecal volvulus can be life-threatening with a mortality rate of 30%. Many studies indicated the time to treatment should be within 24-72 hours of diagnosis [15]. However, even after treatment, the morbidity of the patient is impacted due to wound infection, bowel obstruction, respiratory failure, and prolonged ileus.

The approach to appendicitis causing cecal volvulus is similar to the general management of cecal volvulus and mandates surgical exploration [16].

## Conclusions

In summary, cecal volvulus represents a life-threatening condition that necessitates prompt surgical intervention to mitigate the risk of severe complications such as bowel ischemia and necrosis. The urgency of surgical management cannot be overstated, as delays may exacerbate the patient’s condition and increase the likelihood of adverse outcomes. When determining the appropriate surgical approach, various factors must be carefully considered. The surgeon’s expertise plays a pivotal role, as their proficiency in performing specific procedures influences the overall success of the intervention. Additionally, the stability of the patient must be evaluated, with particular attention paid to hemodynamic status and other critical physiological parameters. Intraoperative findings further guide the decision-making process, providing valuable insights into the extent of bowel involvement and the feasibility of different surgical options. Ultimately, the choice among cecopexy, ileocolic resection, and right hemicolectomy hinges on a comprehensive assessment of these factors, with the primary goal being the restoration of bowel function and the preservation of the patient’s overall health and well-being.

## References

[1] Mohamed AA, Alharbi M, Alrashidi I, Mohamed S (2022). Cecal volvulus a rare cause of intestinal obstruction. A case report. Cureus.

[2] Atamanalp SS, Ozogul B, Kisaoglu A (2012). Cecal volvulus: a rare cause of intestinal obstruction. Eurasian J Med.

[3] Kapadia MR (2017). Volvulus of the small bowel and colon. Clin Colon Rectal Surg.

[4] Cole FR (1956). Volvulus of the cecum associated with acute suppurative appendicitis. N Y State J Med.

[5] Bhatti S, Khan MA, Farooka W, Butt UI, Rehman UA, Malik AA (2017). An unusual case of caecal volvulus due to appendicitis, successfully managed by caecopexy. J Coll Physicians Surg Pak.

[6] Le CK, Nahirniak P, Qaja E (2022). Cecal Volvulus. Cureus.

[7] Le CK, Nahirniak P, Anand S, Cooper W (2022). Volvulus. https://www.ncbi.nlm.nih.gov/books/NBK441836/.

[8] Vogel JD, Feingold DL, Stewart DB, Turner JS, Boutros M, Chun J, Steele SR (2016). Clinical practice guidelines for colon volvulus and acute colonic pseudo-obstruction. Dis Colon Rectum.

[9] Cameron JL, Cameron AM (2022). Current Surgical Therapy, Fourteenth Edition. https://shop.elsevier.com/books/current-surgical-therapy/cameron/978-0-323-79683-5.

[10] Bonadio W (2022). Cecal volvulus in a teenaged patient. J Pediatr.

[11] Gomes CA, Soares C Jr, Catena F, Di Saverio S, Sartelli M, Gomes CC, Gomes FC (2016). Laparoscopic management of mobile cecum. JSLS.

[12] Inberg MV, Havia T, Davidsson L, Salo M (1972). Acute intestinal volvulus. A report of 238 cases. Scand J Gastroenterol.

[13] O'Mara CS, Wilson TH Jr, Stonesifer GL, Cameron JL (1979). Cecal volvulus: analysis of 50 patients with long-term follow-up. Ann Surg.

[14] Majeski J (2005). Operative therapy for cecal volvulus combining resection with colopexy. Am J Surg.

[15] Young WS (1980). Further radiological observations in caecal volvulus. Clin Radiol.

[16] Anderson JR, Welch GH (1986). Acute volvulus of the right colon: an analysis of 69 patients. World J Surg.

